# The current state of knowledge on the role of NKG2D ligands in multiple sclerosis and other autoimmune diseases

**DOI:** 10.3389/fnmol.2024.1493308

**Published:** 2025-01-10

**Authors:** Aleksandra Pogoda-Wesołowska, Nina Sługocka, Agnieszka Synowiec, Klaudia Brodaczewska, Marcin Mejer-Zahorowski, Maciej Ziękiewicz, Wojciech Szypowski, Piotr Szymański, Adam Stępień

**Affiliations:** ^1^Neurology Clinic, Military Institute of Medicine- National Research Institute, Warsaw, Poland; ^2^Faculty of Medicine, University of Warsaw, Warsaw, Poland; ^3^Laboratory of Molecular Oncology and Innovative Therapies, Military Institute of Medicine–National Research Institute, Warsaw, Poland

**Keywords:** multiple sclerosis, NKG2D ligands, review, biomarkers, astrocytes

## Abstract

Multiple sclerosis (MS) is a chronic central nervous system (CNS) disease with demyelinating inflammatory characteristics. It is the most common nontraumatic and disabling disease affecting young adults. The incidence and prevalence of MS have been increasing. However, its exact cause remains unclear. The main tests used to support the diagnosis are magnetic resonance imaging (MRI) examination and cerebrospinal fluid (CSF) analysis. Nonetheless, to date, no sensitive or specific marker has been identified for the detection of the disease at its initial stage. In recent years, researchers have focused on the fact that the number of natural killer cell group 2 member D (NKG2D) family of C-type lectin-like receptor + (NKG2D+) T cells in the peripheral blood, CSF, and brain tissue has been shown to be higher in patients with MS than in controls. The activating receptor belonging to the NKG2D is stimulated by specific ligands: in humans these are major histocompatibility complex (MHC) class I polypeptide–related sequence A (MICA) and MHC class I polypeptide-related sequence B (MICB) proteins and UL16 binding 1–6 proteins (ULBP1-6). Under physiological conditions, the aforementioned ligands are expressed at low or undetectable levels but can be induced in response to stress factors. NKG2D ligands (NKG2DLs) are involved in epigenetic regulation of their expression. To date, studies in cell cultures, animal models, and brain tissues have revealed elevated expression of MICA/B, ULPB4, and its mouse homolog murine UL16 binding protein-like transcript (MULT1), in oligodendrocytes and astrocytes from patients with MS. Furthermore, soluble forms of NKG2DLs were elevated in the plasma and CSF of patients with MS compared to controls. In this review, we aim to describe the role of NKG2D and NKG2DLs, and their interactions in the pathogenesis of MS, as well as in other autoimmune diseases such as rheumatoid arthritis (RA), inflammatory bowel disease (IBD), systemic lupus erythematosus (SLE), and celiac disease (CeD). We also assess the potential of these proteins as diagnostic markers and consider future perspectives for targeting NKG2D ligands and their pathways as therapeutic targets in MS.

## 1 Introduction

Multiple sclerosis (MS) is a chronic demyelinating inflammatory disease of the central nervous system (CNS). There are an estimated 2.1–2.2 million people with MS worldwide, and the disease is approximately two to three times more common in women than in men, which is related to estrogen levels (Ebers, 2005). According to the European Multiple Sclerosis Platform (EMSP), more than 700,000 individuals have MS in Europe. The onset of MS typically begins between the ages of 20–40 years, which is at the time of peak labor force participation and family formation. It is the most common non-traumatic disabling disease affecting young adults. The disease shortens life expectancy by an average of 6–7 years. Death can occur due to disease-related complications associated with neurological symptoms and immobilization. The incidence and prevalence of MS are increasing in both developed and developing countries. However, its exact cause remains unclear (Browne et al., 2014). It is a multifactorial autoimmune disorder, with multifocal CNS damage at its core. To date, several genetic and environmental factors have been identified that may slightly increase susceptibility to this disease, particularly vitamin D deficiency or exposure to ultraviolet B (UVB) radiation, Epstein–Barr virus (EBV) infection, obesity, and smoking (Ascherio, 2013).

The multifactorial etiology of MS, the multiplicity of pathological processes in the course of the disease, and its various forms hinder the diagnostic process and delay final diagnosis. The main tests used to support the diagnosis are magnetic resonance imaging (MRI) and cerebrospinal fluid (CSF) analysis (Hauser and Cree, 2020). To date, no sensitive or specific marker has been found to detect the disease in its early stage. New markers and diagnostic tests to allow the rapid and easy detection of the disease and the implementation of appropriate treatment before the onset of clinical symptoms are currently being investigated.

**Figure 1:**
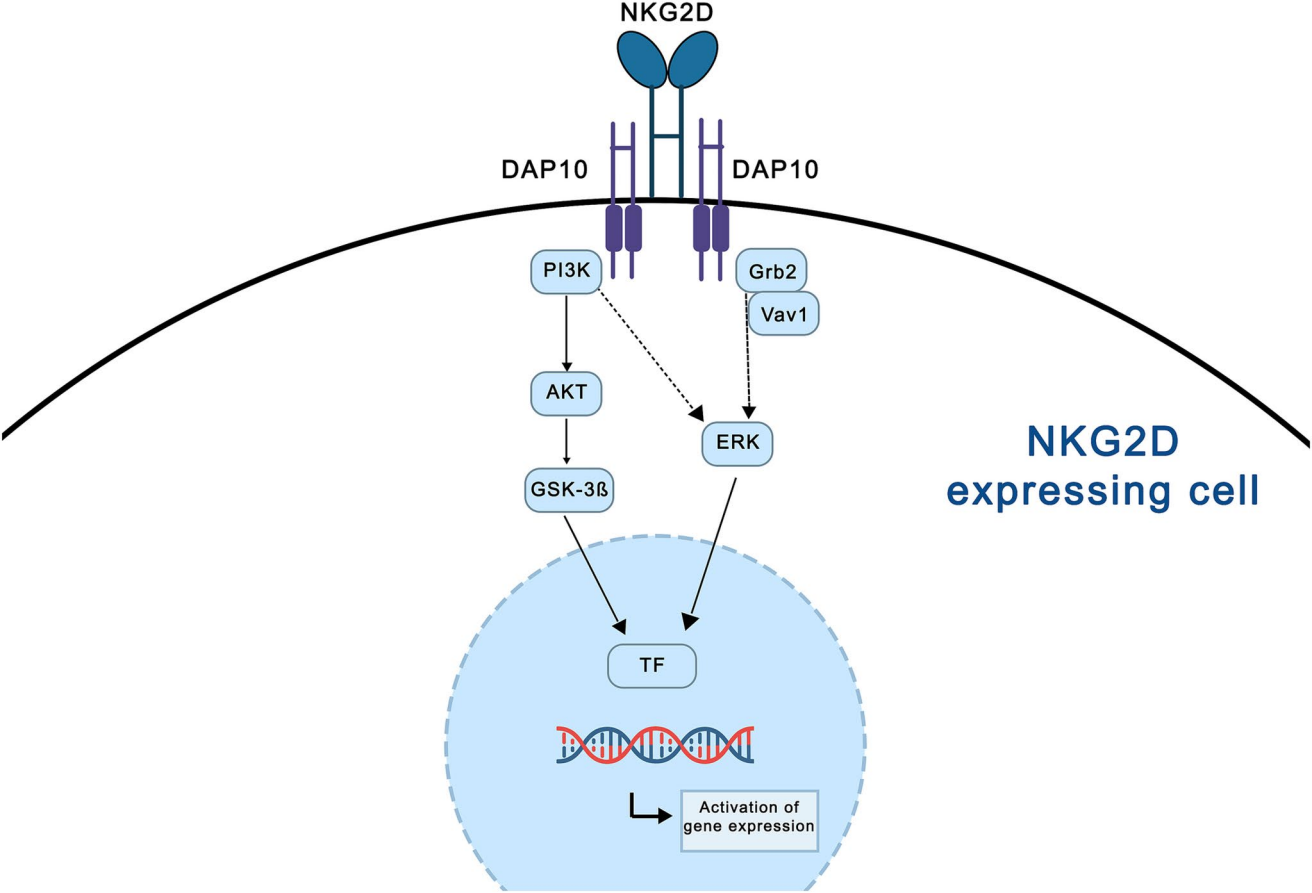
Schematic representation of NKG2D main signaling pathways in human NK cells. NKG2D forms an activating complex upon association with the transmembrane protein DAP10, which activate the PI3K-AKT-GSK-3β and Grb2-ERK via Vav1 signaling pathways. Activation of PI3K pathway initiating ERK kinase and is necessary to the phosphorylation and activation of AKT which leads to the phosphorylation of GSK-3β. Grb2 induces activation of ERK kinase. These signaling pathways are required e.g. to promote cell growth, transcription, activation of gene expression and cell proliferation. This figure was created using Adobe Photoshop. NKG2D - natural killer cell group 2 member D; DAP10 – DNAX- activating protein 10; PI3K: phosphatidylinositol 3-kinase; AKT: protein kinase B; GSK-3β: glycogen synthase kinase 3 β; Grb2: growth factor receptor-bound protein 2; Vav1: *vav* guanine nucleotide exchange factor 1; ERK: extracellular signal-regulated kinase; TF- transcription factors.

Natural killer (NK) and T cells are important types of innate and adaptive immune cells. The responses of NK and T cells to pathogens and tumors are regulated by signals from various receptors expressed on their cell surfaces, which can initiate, enhance, or inhibit effector cell function (Tan et al., 2023). NK cells are mainly determined by the activating and inhibitory receptors on their surfaces (Zhang et al., 2023). Most NK receptors are also expressed on CD8+ and CD4+ T cells (Tan et al., 2023). Therefore, the aberrant expression of NK cell receptor ligands on target cells can induce NK- or T-cell-dependent autoimmune responses (Babic and Romagnani, 2022). NK cell group 2 member D (NKG2D) is one of the best-characterized receptors shared by NK and T cells (Alkhayer et al., 2023). In humans, there are two families of NKG2D ligands (NKG2DLs), major histocompatibility complex (MHC) class I chain-related proteins (MIC) and UL16-binding proteins (ULBP) families (Wu et al., 2021). Mouse NKG2DLs consist of members of the mouse UL-16-binding protein transcript-like 1 (MULT1), retinoic acid early inducible (RAE)-1α-*ε*, and tissue compatibility protein 60 (H60) a-c families (Wu et al., 2021). In addition, NKG2DLs are attached to the membrane in various ways, with some being transmembrane proteins, such as MIC, ULPB4, ULBP5, Mult-1, H60a, and H60b, and others being anchored proteins, such as ULBP1–3, ULBP6, H60c, and RAE-1, attached to the membrane via glycosylphosphatidylinositol (GPI) anchors (Duan et al., 2019). NKG2DLs are stress proteins that generally show low expression in normal cells and prevent the development of autoimmune diseases, except in the presence of cytokines, viral infections, oxidative stress, ionizing radiation, and DNA damage, where their expression increases (Lee and Blish, 2023). The downstream mechanisms of NKG2D activation are shown in Figure 1.

It is hypothesized that the levels of soluble forms of selected NKG2DLs in the CSF and plasma of patients with MS are higher than those in control patients. For example, in a study by Zingoni et al. (2018) soluble forms of MHC class I chain-related protein sequence A (MICA) and sequence B (MICB) were detected in the plasma of patients with MS. However, no data have been obtained regarding their presence in the CSF. However, increased levels of the soluble form of UL 1–6 binding protein 4 (sULBP4) in the CSF have been observed in women with MS compared to men and controls (Saikali et al., 2007).

Moreover, in a study by Dai et al. (2009) of adolescent patients with systemic lupus erythematosus (SLE), an increased frequency of soluble MICA (sMICA) and soluble MICB (sMICB) was observed in the peripheral blood, which was negatively correlated with SLE severity. Furthermore, NKG2D expression on the surface of NK cells is lower in patients with SLE than that in healthy controls (Green et al., 2005). Similarly, increased levels of sMICA have been reported in the peripheral blood of patients with rheumatoid arthritis (RA) (Groh et al., 2003; Dai et al., 2009). Additionally, sMICA/sMICB reduces NKG2D expression on the surface of NK cells and impairs NK cell function, allowing cancer cells to evade NKG2D-dependent immune surveillance (Duan et al., 2019). Moreover, studies have shown that MICA is significantly upregulated in the intestinal epithelial cells of patients with Crohn’s disease (CD) and ulcerative colitis (UC) and may participate in the pathogenesis of inflammatory bowel disease (IBD) via NKG2D–MICA interactions (Allez et al., 2007; Ge et al., 2011; Muro et al., 2014; Espinoza and Minami, 2018). In celiac disease (CeD), MICA/B expression in intestinal mucosa is associated with impaired mucosal homeostasis (Allegretti et al., 2013).

It is possible that the levels of soluble forms of NKG2DLs in the plasma reflect their levels in the CSF; however, this topic requires further study. If the assumption was confirmed, it would indicate the possibility of a new peripheral biomarker in a fast and easily accessible manner without the need for lumbar puncture. Such a marker is desirable in both MS and other inflammatory diseases, and the use of the described NKG2DLs in this role is worth examining, given the findings reported above.

In the present review, we summarize the roles of NKG2D and NKG2DLs and their interactions in the pathogenesis of MS, as well as their involvement in the pathophysiology of other autoimmune diseases, such as RA, IBD, SLE, and CeD. We also assess the potential of these proteins as diagnostic markers and consider the future perspectives for targeting NKG2DLs and their pathways as therapeutic targets in MS.

## 2 Role of astrocytes in MS pathobiology

Increasing evidence supports the key role of reactive astrocytes in the pathobiology of MS at different stages (Brambilla, 2019). Astrocytes respond to CNS diseases and injury through cell proliferation, profound morphological changes (e.g., increased branching, increased cell size, and cell elongation), and functional modifications. This phenomenon, referred to as astrogliosis, has both positive and negative effects on the neurological system (Escartin et al., 2021). As the processes activated in reactive astrocytes during CNS autoimmunity are both detrimental and reparative, the roles of these cells in disease pathophysiology are complex. Astrocytes are frequently detected in lesions present in MS (Lassmann, 2018). They are regulated by inflammatory mediators that not only induce glial cell reactivity, but also lead to cellular stress and oxidative damage (Pekny et al., 2016). In MS, astrocytes undergo cellular activation, which is evident in the early disease stages. This causes astrocytes to gain the ability to produce multiple soluble mediators, both neurotoxic and neuroprotective, and lose the ability to maintain homeostatic functions important for CNS integrity. Astrocytes produce oxidants, cytokines, and chemokines that enhance immune activation, thereby causing tissue damage. The key role of astrocytes in shaping local inflammatory responses by interacting with other nerve cells and infiltrating leukocytes is currently under investigation (Brambilla, 2019). Owing to their neuroprotective capacity, astrocytes produce anti-inflammatory and promyelinating factors, and prevent CNS invasion by immune cells through glial scar formation. Gliosis, non-neoplastic hypertrophy, and proliferation of stellate glial cells (astroglia) are non-specific responses of astrocytes (Pekny et al., 2016). To date, studies have provided evidence of a positive correlation between astroglial activation and the severity and evolution of MS. This suggests that strategies aimed at the general suppression of astroglial activation may be effective in reducing MS symptoms. In contrast, measuring astroglial activation in patients may provide a means to monitor disease severity, predict disease progression, and test the efficacy of MS medications.

## 3 The role of NKG2D receptors and their ligands in MS and other diseases

In recent years, researchers have focused on the fact that all infiltrating CD8+ T lymphocytes present in demyelinating plaques in MS express NKG2D receptors (Ruck et al., 2013). NKG2D is an activating receptor belonging to the NKG2 family of C-type lectin-like receptors expressed by immune effector cells, including NK cells, and CD8+ and CD4+ T cell subsets. NKG2D stimulates many immune processes, including co-stimulation of T cells, thereby increasing their cytotoxicity, chemokine responsiveness, and inflammatory cytokine production (Wensveen et al., 2018). It has been found that these ligands, which are normally increased in response to cellular stress, are important in several disorders other than MS. Several cellular stressors present in chronic inflammatory and autoimmune diseases have been shown to increase the expression of specific NKG2DLs, resulting in the activation of immune effector cells containing NKG2D (Babic and Romagnani, 2018).

The diverse range of circumstances in which soluble NKG2DLs affect tumor growth and immune responses underscores the intricate nature of their functions, including cancer biology (Zingoni et al., 2018; Wensveen et al., 2018). The aberrant expression of NKG2DLs has been implicated in several autoimmune diseases as well (Groh et al., 2003; Babic and Romagnani, 2018).

The involvement of NKG2D and its ligands in autoimmune diseases was first revealed in RA, but has also been described in type I diabetes (Ogasawara et al., 2004; Blevins et al., 2012; Babic and Romagnani, 2018). T lymphocytes expressing NKG2D are present in the foci of chronic inflammatory diseases, such as active skin lesions in vitiligo, intestinal epithelium in CeD, intestinal lamina propria in CD, and synovial membrane in RA, and are associated with the production of inflammatory mediators (Ogasawara et al., 2004; Babic and Romagnani, 2018; Jacquemin et al., 2020). Previous studies have shown that the number of NKG2D+ T lymphocytes in the blood, CSF, and brain tissue of patients with MS is higher than that in controls (Ruck et al., 2013).

NKG2D interacts with different ligands: in humans there are MICA and MICB, and ULBP1-6; in mice they include RAE-1-*α*, −*β*, −*γ*, −*δ*, and −*ε*; H60a, H60b, H60c; and mouse Mult-1 (Zingoni et al., 2018). Under physiological conditions, the aforementioned ligands are expressed at low or undetectable levels but can be induced in virtually any cell in response to stressors such as DNA damage, inflammation, oxidative stress, endoplasmic reticulum stress, or oncogenic transformation, which acts as an alert for the immune system (Zingoni et al., 2018). NKG2DLs are subjected to transcriptional, post-transcriptional, translational, and post-translational regulatory mechanisms that modulate their expression (Raulet et al., 2013). Tight regulation of the expression of these ligands is crucial for controlling the activation of the NKG2D-NKG2DL pathway. It is increasingly being inferred that pathologically elevated NKG2DL levels may contribute to chronic inflammatory and autoimmune diseases (Hüe et al., 2004; Allez et al., 2007; Allez et al., 2017). This is supported by studies that have described higher NKG2DL expression in the intestines of patients with CD and CeD than in healthy donors (Hüe et al., 2004; Allez et al., 2007). Furthermore, the possibility of targeting therapeutic treatments to the NKG2D pathway in human inflammatory diseases was confirmed by administering a single injection of *α*-NKG2D neutralizing antibody to patients with Crohn’s disease, which reduced disease activity (Allez et al., 2017). The main characteristics of these proteins are summarized in Table 1.

**Table 1:** NKG2D ligands overview.

Protein	Domain organization	Gene location	Anchoring mechanism	Species specificity	Research in MS/EAE	Research in other diseases
MICA/B	α1, α2, and α3 domains	Chromosome 6 within MHC locus	TM	Human	Yes (40)	Cancers (Salih et al., 2006), RA (Babic and Romagnani, 2022), SLE (Groh et al., 2003), IBD (Allez et al., 2007), CeD (Hüe et al., 2004), rejection in organ transplantation (Suarez-Alvarez et al., 2006)
ULBP1	α1 and α2 domains	Chromosome 6 outside MHC locus	GPI-anchored	Human	Not extensive	Cancers including HCC (Easom et al., 2020), viral infections, IBD (Allez et al., 2007)
ULBP2	α1 and α2 domains	Chromosome 6 outside MHC locus	GPI-anchored	Human	Not extensive	Cancers (breast, ovarian (Kamimura et al., 2012), pancreatic), CMV (Cosman et al., 2001)
ULBP3	α1 and α2 domains	Chromosome 6 outside MHC locus	GPI-anchored	Human	Not extensive	Cancers including HCC (Easom et al., 2020), CMV (Cosman et al., 2001)
ULBP4	α1 and α2 domains	Chromosome 6 outside MHC locus	TM	Human	Yes (46)	Cancers (Zöller et al., 2018), RA (Zingoni et al., 2018)
ULBP5	α1 and α2 domains	Chromosome 6 outside MHC locus	TM	Human	Not extensive	Cancers including HCC and lung cancer (Easom et al., 2020), CMV (Nice et al., 2009)
ULBP6	α1 and α2 domains	Chromosome 6 outside MHC locus	GPI-anchored	Human	Not extensive	Cancers (Kamimura et al., 2012; Zingoni et al., 2018)
MULT1	α1, α2, and α3 domains	Chromosome 10	TM	Mouse	Yes (49)	Cancers (Zhang and Sentman, 2013), MCMV (Nice et al., 2009)
RAE-1	α1 and α2 domains	Chromosome 10	TM	Mouse	Yes (51)	Cancers (Lam et al., 2014), MCMV (Hasan et al., 2005)
H60	α1 and α2 domains	Chromosome 10	TM	Mouse	Yes (54)	Cancers (O'Callaghan et al., 2001), MCMV (Hasan et al., 2005)

CeD, celiac disease; CMV, cytomegalovirus; EAE, experimental autoimmune encephalomyelitis; HCC, human hepatocellular carcinomas; GPI, glycosylphosphatidylinositol; IBD, inflammatory bowel disease; MCMV, mouse cytomegalovirus; MHC, major histocompatibility complex; MS, multiple sclerosis; RA, rheumatoid arthritis; SLE, systemic lupus erythematosus; TM, transmembrane.

### 3.1 MICA and MICB

MICA and MICB are encoded in the MHC region and share structural and sequence similarities with HLA class I genes (28–35%). Under physiological conditions, their expression is restricted to different areas of the intestinal epithelium with limited surface expression (Groh et al., 1998). However, their expression levels increase in response to ill-defined stressors, such as cancer transformation, infection, hypoxia, heat shock, and oxidative stress. MICA is a member of the nonclassical class I family and displays the greatest degree of polymorphism. MICA alleles can be divided into two large groups with the polymorphisms found in α3 domains. MICA polymorphisms are associated with several diseases related to NK activity such as viral infections, cancer, allograft rejection, and graft-versus-host disease. The mechanisms underlying these associations include NK cell-mediated cytotoxicity and MICA shedding to produce soluble immunosuppressive MICA particles (Choy and Phipps, 2010). MICB is related to MHC class I and has similar domain structure, which is made up of an external α1α2α3 domain, transmembrane segment and C-terminal cytoplasmic tail (Bahram et al., 1994). MICB is a stress-induced ligand of the NK cell-activating receptor NKG2D, and is critical for the NK cell killing virus-infected and tumor cells. In a study conducted by Stern-Ginossar et al. (2007) human cytomegalovirus-miR-UL112 specifically downregulated MICB expression during viral infection, leading to decreased binding of NKG2D and reduced killing by NK cells. These results suggest that human cytomegaloviruses exploit a miRNA-based immunoevasion mechanism.

In an experiment conducted on brain tissues collected postmortem from patients with MS, oligodendrocytes expressing MICA and MICB were demonstrated, and CD8+ T cells were observed in close proximity to them, suggesting possible contact between T cells and NKG2DL+ cells in MS (Saikali et al., 2007). In a cell culture study, the authors showed that NKG2DLs are present on human oligodendrocytes and that the disruption of NKG2D-NKG2DL interactions inhibits the destruction of human oligodendrocytes via activated human immune effector cells, including CD8+ T cells (Saikali et al., 2007). In a subsequent study, a soluble form of MICA and MICB was found to be present in the plasma of patients with MS, but no data are available on its presence in the CSF (Zingoni et al., 2018). Fernández-Morera et al. (2008) analyzed serum and tissue (normal-appearing white matter and lesional white matter) samples from 40 patients with relapsing–remitting MS (27 women and 13 men; mean age, 34 years) and compared them with a control group, which consisted of 11 healthy blood donors. Most patients were being administered interferons, and none used other immunomodulatory or immunosuppressive drugs. It was observed that sMICB levels in the sera of patients with MS were significantly higher than those in healthy controls (128 vs. 40 pg./mL; *p* < 0.01). In addition, the serum sMICB concentration in the MS group was higher during the clinical relapse than during the remitting phase of the disease (161 vs. 73 pg./mL; p < 0.01). Thus, sMICB appears to be a useful marker of disease activity. No statistically significant differences were observed between serum sMICA levels in patients with MS and controls. Moreover, this study demonstrated that MIC is expressed in astrocytes and neurons with an intracellular distribution during inflammatory activity in MS. This finding suggests that MICA and MICB can be released in soluble form to modulate NKG2D-related cytotoxic activity in NKG2D-positive cells. The presence of intracellular stored amounts of MIC molecules in the CNS neurons of MS pathological samples could be responsible for the high soluble MICB levels during relapses if these molecules were to be released. Further studies are necessary to determine whether corticosteroid treatment affects serum MICB levels during the recovery from relapse.

In another study conducted by Fernandez-Morera et al. (2008) the MICB*004 allele frequency was significantly increased in patients with MS (46.3 vs. 23.3%, Pc < 0.001, odds ratio 2.82, 95% confidence interval 68–4.73), and only the frequency of the HLA-DRB1*01 allele was increased in controls (31 vs. 14%, Pc = 0.011). This suggests that MHC class I molecules, in addition to class II molecules, may be involved in MS susceptibility. Similarly, Abediankenari et al. (2011) found that MICB gene expression was higher in the peripheral blood mononuclear cells collected from 32.6% of patients with progressive MS compared to controls (*p* = 0.002). Moreover, Graves et al. (2014) noted an association between DNA methylation of HLA-DRB1 and MS risk.

In contrast, sMICB has recently been reported to impair the immunogenicity of tumors by reducing NKG2DL density in malignant cells (Salih et al., 2006). Moreover, the presence of soluble MIC molecules has also been described in RA, shed by RA synoviocytes, leading to the activation of autoreactive T cells, exacerbating inflammation and joint damage, and in the serum of heart transplant individuals, associated with a lower incidence of rejection (Groh et al., 2003; Suarez-Alvarez et al., 2006). Additionally, we investigated the expression of NKG2DLs in the intestinal epithelium of patients with CeD and its premalignant complication, refractory sprue. This study showed that stress-induced upregulation of the MICA/NKG2D pathway might lead to the activation of intraepithelial lymphocytes, resulting in epithelial damage (Hüe et al., 2004). In addition, the MICA-induced humoral response has recently attracted interest because of its possible role in graft rejection during solid organ transplantation. High levels of soluble MICA are associated with graft acceptance after heart transplantation (Fernandez-Morera et al., 2008).

### 3.2 UL 1–6 binding proteins

UL 1–6 binding proteins (ULBPs), also referred to as retinoic acid early transcripts (RAETs), represent a distinct family of ligands for human NKG2D receptors. These proteins are expressed across a spectrum of immune cells, including NK cells, CD8+ αβ and γδ T cells, and a subset of CD4+ T cells (Fernández-Messina et al., 2012). In contrast to MICA/B, ULBPs are encoded outside the MHC locus on chromosome 6. Notably, ULBPs are partially characterized as glycosyl-phosphatidyl-inositol (GPI)-anchored molecules, diverging from the transmembrane (TM) proteins MICA and MICB. This discrepancy in membrane anchoring implies disparate biosynthesis and trafficking pathways within cells (Blevins et al., 2012). ULBPs structurally resemble traditional MHC class I molecules with their α1 and α2 domains; however, they are not associated with β2-microglobulin and are not involved in antigenic peptide presentation. Six different genes, including ULBP 1–6, have been identified in the ULBP family, and their amino acid sequences are approximately 55–60% similar (Fernández-Messina et al., 2012).

#### 3.2.1 ULBP1

UL 1–6 binding protein 1 (ULBP1) is a GPI-linked member of the ULBP family. ULBP1 selectively interacts with NKG2D receptors, which are expressed on NK cells, CD8+ T cells, and some subsets of γδ T cells (Raulet et al., 2013). Stressed or altered cells expressing ULBP1 are eliminated as a result of this interaction, resulting in cytotoxic reactions (Bauer et al., 1999). These immune cells are activated upon ULBP1 binding to NKG2D, which increases their cytotoxic activity and cytokine production. This aids in immune surveillance and removal of weakened, infected, or cancerous cells. Elevated ULBP1 levels have been observed under various conditions including cellular stress, infection, malignancy, and autoimmune diseases. For instance, elevated ULBP1 levels can serve as an indicator of cellular stress or tumorigenesis. These cells may serve as targets for NK cell-mediated lysis because of their overexpression. However, ULBP1 may be secreted by certain tumor cells and released into the surrounding environment. By suppressing NK cell production of NKG2D, soluble ULBP1 may aid malignancies in immune monitoring (Raulet et al., 2013).

#### 3.2.2 ULBP2

Another GPI-linked part of the ULBP family is UL 1–6 binding protein 2 (ULBP2), which is a ligand for the NKG2D receptor on NK cells and some T cells. However, ULBP2 can also exist in a soluble form without a GPI anchor because of alternative splicing or proteolytic shedding, which can lead to different immunomodulatory effects (Raulet et al., 2013). Normal cells typically produce very low levels of ULBP2. Its expression is thought to be a response to cellular stressors such as heat shock, DNA damage, and infection. Specific signaling pathways have unique regulatory effects on ULBP2. More than other ULBPs, it is highly induced by DNA damage via the ATM/ATR pathway (Raulet et al., 2013). Moreover, ULBP2 has higher binding affinity for the NKG2D receptor than most other ULBPs, which is associated with more robust immune activation (Oppenheim et al., 2005).

#### 3.2.3 ULBP3

UL 1–6 binding protein 3 (ULBP3) belongs to the ULBP family and is GPI-linked, indicating that ULBP3s are generally membrane-bound (Raulet et al., 2013). ULBP3 is not commonly found on the surface of most normal cells. Its expression is stimulated by cellular stressors, such as infection, transformation (cancer), or other pathological conditions. Heat shock, genotoxic stress, and DNA damage response pathways can all increase the expression of ULBP3 (Raulet et al., 2013). NK cells and certain subsets of T cells are activated when ULBP3 binds to the NKG2D receptor. The ability of the immune system to identify and eradicate strained, infected, or cancerous cells critically depends on this connection. Like other NKG2D ligands, ULBP3 plays a role in immune surveillance and the removal of potentially harmful cells by the immune system (Lanier, 2015).

#### 3.2.4 ULBP4

A poorly studied human NKG2D ligand, UL 1–6 binding protein 4 (ULBP4) is a unique member of the ULBP family that differs from other members of the ULBP family in a number of ways. Although many ULBPs (such as ULBP1, ULBP2, and ULBP3) are attached to the cell membrane via a GPI anchor, ULBP4 is a transmembrane protein. This difference affects its biosynthesis, trafficking, and perhaps its interactions with other cellular proteins (Oppenheim et al., 2005).

ULBP4 is encoded by the retinoic acid early transcript 1E (RAET1E) locus in the centromeric region of the ULBP gene cluster on the long arm of human chromosome 6 (Zöller et al., 2018). To identify the relatives of the ULBP family members ULBP1, ULBP2, and ULBP3, which had previously been identified during a search for binding partners of the human betaherpesvirus 5 glycoprotein UL1-6 and were named accordingly, in silico screens of human genomic sequences were used to identify ULBP4 (Cosman et al., 2001). The cDNA of ULBP4 encodes a polypeptide with 263 amino acids, including a signal peptide. This polypeptide matures into a 235 amino acid (approximately 27 kDa) protein that is attached to the cell surface. There are four exons that encode this ULBP4 polypeptide: exon 1 encodes the signal peptide, exon 2 the *α*1 domain, exon 3 the α2 domain, and exon 4 the transmembrane region, the short serine-rich stalk, and a short cytoplasmic domain (Zöller et al., 2018).

Among the proteins in the ULBP family, ULBP4 is the most polymorphic. Some malignancies, EBV-infected B cells, and cytokine-activated NK cells have been reported to produce ULBP4, which may be important for NKG2D-mediated immunosurveillance and immunoregulation. The characterization of ULBP4 glycoproteins is unsatisfactory, and there are significant discrepancies between published research concerning aspects such as biochemical characteristics and tissue and cell line expression (Zöller et al., 2018).

ULBP4 enhances the cytotoxic potential of NK cells against target cells, including tumor cells, by inducing NK cells to generate cytokines and chemokines through its interaction with NKG2D. Because of this connection, the inhibitory signals of MHC class I molecules may be overridden, leaving ULBP4-expressing cells vulnerable to NK cell-mediated destruction (Cosman et al., 2001). Moreover, in contrast to other ULBPs, ULBP4 is believed to be expressed on healthy monocytes. Monocyte ULBP4 regulates the activity NKG2D in NK cells (Sharma and Markiewicz, 2019). It has been suggested that ULBP4 should not be considered just as another NKG2DL, but rather as having distinct cellular expression and processing, along with its early evolutionary split from other members of the ULBP family, pointing to an exclusive function for ULBP4 within the context of NKG2D-mediated surveillance of immunity (Zöller et al., 2018).

#### 3.2.5 ULBP5

Regarding the placement of genes and the structure of proteins, UL 1–6 binding protein 5 (ULBP5) resembles other ULBPs. ULBP5 may, however, have distinct qualities that distinguish it from other ULBPs owing to particular structural elements or sequence modifications. The α domain layout is one such example. Although ULBPs typically have two exterior domains (α1 and α2), their interaction with the NKG2D receptor might vary depending on the precise amino acid sequence and the resulting three-dimensional structure (Eagle et al., 2009). Compared to ULBP4, ULBP5 is a transmembrane component of the UL 1–6 binding protein family (Raulet et al., 2013).

Targeting and eliminating agitated or altered cells is what ULBP5 does when it attaches to the NKG2D receptor in NK cells and certain T cells. Although different ULBPs, including ULBP5, exhibit varied affinities and kinetics in their binding to NKG2D, it is possible that the binding kinetics and affinity of ULBP5 to NKG2D differ from those of other ULBPs, which could impact the duration and strength of the immune response (Cosman et al., 2001). Several signaling pathways, such as those activated by viral infection, DNA damage, and other types of cellular stress, control the expression of ULBP5, which may have different regulatory routes and pathways with different levels of efficiency.

#### 3.2.6 ULBP6

UL 1–6 binding protein 6 (ULBP6) is comparatively similar to other members of the ULBP 1–6 family. Similar to most ULBPs, ULBP6 is a GPI-anchored protein, which differentiates it from ULBP 4 and ULBP5 (Raulet et al., 2013). Its protein structure and expression patterns do not differ from those of other ULBPs. In line with other ULBPs, ULBP6 has two exterior domains (α1 and α2) that aid in its interaction with the NKG2D receptor. Its expression is also elevated in response to cellular stress, although it may have unique expression profiles in different tissues.

However, a noteworthy characteristic that differentiates ULBP6 from other ULBPs is its high level of polymorphism. Significant genetic diversity of ULBP6 may affect its immunological recognition and binding affinity. This polymorphism was not as noticeable in other members of the ULBP family. High polymorphism can improve the capacity of the immune system to identify a wide variety of infections and stress signals (Fernández-Messina et al., 2012). The fact that ULBP6 is polymorphic may also pose an issue. This increases the range of signals that the immune system can recognize but also suggests that some polymorphic variants may be more adept at eluding immune identification.

#### 3.2.7 ULBP1-6 in MS

ULBP1-6, as ligands for the NKG2D receptor, play a significant role in the pathogenesis of MS by enhancing the activation and recruitment of immune cells to the CNS. ULBPs and other NKG2D ligands are upregulated in MS lesions. Inflammatory processes linked to MS are facilitated by increased NKG2D+ CD4+ T cell activation. Elevated expression of these ligands in MS lesions implies that they may be involved in the activation and recruitment of autoreactive T cells, which in turn may contribute to the autoimmune pathology observed in patients with MS. This upregulation of ULBPs under these conditions promotes the activation of immune cells via the NKG2D receptor, contributing to an autoimmune attack on myelin (Saikali et al., 2007).

Of all the ULBP family members, only ULBP4 has been studied extensively to draw conclusions about its effects on MS. Elevated ULBP4 protein expression has been described in brain tissue collected post-mortem from patients with MS, and astrocytes were the cell type most commonly expressing this ligand (Moratalla et al., 2022). Additionally, it has been demonstrated that stressors present in MS can induce ULBP4 expression by human astrocytes (Moratalla et al., 2022). In cell culture studies, inflammation, endoplasmic reticulum stress, and oxidative stress increased ULBP4 expression in human astrocytes, resulting in NKG2D-mediated destruction by activated NK cells. It is noteworthy, that at the same time the expression of this ligand in human neurons did not change. In addition, it has been shown that ULBP4 protein expression is increased in astrocytes surrounding blood vessels, suggesting that NKG2D+ immune cells passing through, which include all infiltrating CD8+ T cells and a subset of CD4+ T cells, may encounter astrocytes with ULBP4 expression (Bauer et al., 1999). Increased levels of the soluble form of ULBP4 in the CSF of female patients with MS compared to those in male patients with MS and controls have also been reported (Moratalla et al., 2022). Furthermore, soluble ULBP4, like MULT1 in mice, has been shown to enhance the secretion of inflammatory cytokines by CD8+ T cells, mainly granulocyte-macrophage colony-stimulating factor (GM-CSF) and interferon (IFN)-*γ* (Zingoni et al., 2018). It is also possible that the presence of soluble ULBP4 enhances T cell movement in the brain parenchyma via non-chemotactic mechanisms.

The role of ULBPs in the context of MS remains relatively unexplored, rendering it a particularly intriguing and promising area of research. The paucity of research on the subject to date emphasizes the need for a deeper understanding of the role of these proteins in the etiology and development of MS. ULBPs may play a major role in the autoimmune mechanisms that underlie MS owing to their involvement in immune surveillance and their interaction with the NKG2D receptor on NK cells and some T cells. Further research is needed on the differential expression and regulatory mechanisms of ULBPs in patients with MS. Such studies could elucidate the unique contributions of individual ULBP family members to disease progression. This deeper understanding may ultimately lead to the identification of novel therapeutic targets, thereby improving treatment strategies and clinical outcomes in patients with MS.

In recent years, the NKG2D pathway has attracted interest as a possible target for cancer therapy, particularly ULBPs. Several studies have linked a better prognosis to ULBP1 expression on tumor cells in the context of cancer. These findings suggest improved immune recognition and possible reactivity to NKG2D pathway-targeting immunotherapy. Certain cancerous cells, such as human hepatocellular carcinoma (HCC) cells, contain ULBP1 as a cell surface protein. As ULBP1 is widely expressed in dysplastic nodules in moderately differentiated HCC, tumor development and early recurrence are associated with its decreased expression (Kamimura et al., 2012). Moreover, serum ULBP1 levels were high, which may indicate that HCC shedding of this ligand into the bloodstream caused the observed cell surface ULBP1 loss. Additionally, soluble ULBP1 is produced when HCC occurs but not when other cancers metastasize to the liver, indicating its diagnostic specificity. Although blood ULBP1 was significantly higher in patients with HCC, its applicability as an HCC screening tool was diminished by the fact that it was also detectable to varying degrees in HBV-related diseases without HCC (Cosman et al., 2001). Other proteins are more important in HCC than ULBP1. Studies have been conducted on the potential significance of ULBP3 in predicting recurrence of HCC following radical resection. NKG2D ligand expression at the mRNA and protein levels is associated with recurrence-free survival, and ULBP3 is an important predictive factor. ULBP3 expression is notably associated with other NKG2DLs, which suggests an important role for ULBP3 in controlling the expression of other NKG2DLs (Chen et al., 2023). Given its lower expression in tumor tissues than in adjacent normal tissues, strategies aimed at upregulating ULBP3 could be explored as a therapeutic approach to enhance the immune recognition and destruction of tumor cells. Furthermore, in addition to MICA and ULBP3, ULBP5 may be helpful in predicting the recurrence of HCC in patients after hepatectomy and may be a good predictor of postoperative patient management (Carayannopoulos et al., 2002). One key element in cancer is the ULBP5 ligand. Tumor tissues exhibit considerably higher mRNA expression level of ULBP5 in comparison to nearby normal tissues, suggesting that ULBP5 is upregulated in the tumor microenvironment (Carayannopoulos et al., 2002).

### 3.3 MULT1

Murine UL16-binding protein-like transcript 1 (MULT1) is an essential ligand for the NKG2D receptor and functions as an important mediator of the immune response by indicating cellular stress or transformation. This ligand is exclusive to mice and does not have a direct human homolog. MULT1 plays a crucial role in the activation of T-lymphocyte subsets and NK cells. The single-pass transmembrane domain of MULT1, a type I transmembrane protein, has an N-terminus that is oriented extracellularly and a C-terminus intracellularly (Chen et al., 2023). An immunoglobulin-like domain, which is characteristic of proteins involved in immunological signaling, has been identified. This domain is intended to permit high-affinity binding and enhance interactions with the NKG2D receptor. MULT1 exhibits a higher affinity for the NKG2D receptor than other murine ligands, such as RAE-1 and H60. This high affinity is essential for robust NK cell activation and an efficient immune response, even when MULT1 is expressed at low levels (Diefenbach and Raulet, 2002). The unique regulatory mechanism of MULT1 involves post-translational modifications via ubiquitination. Under non-stressed conditions, MULT1 is ubiquitinated and undergoes proteasomal degradation. The accumulation of MULT1 on the surface of healthy cells is inhibited by this regulation. Stress reduces ubiquitination, which maintains MULT1 on the cell surface and enables it to interact with NKG2D receptors (Nice et al., 2009).

It has been reported that levels of the soluble form of mouse MULT1 protein are elevated in the CSF of mice with experimental autoimmune encephalomyelitis (EAE), which may enhance the inflammatory properties of CD8+ T cells (Legroux et al., 2019). EAE is a model of MS characterized by microglial proliferation and activation, immune cell recruitment, and neurogenesis. It has been observed that MULT1 protein levels are elevated in the mouse CNS during MS relapse, whereas levels of the soluble form of MULT1 are elevated in the CSF during both active and passive EAE (Legroux et al., 2019). Furthermore, it has been shown that the soluble form of MULT1 enhances the effector functions (e.g., IFN-*γ* production) of activated CD8 NKG2D+ T cells (Legroux et al., 2019). It has also been reported that blocking NKG2D leads to a significant reduction in the severity of clinical symptoms and regression of pathological changes induced by myelin oligodendrocyte peptide in EAE. NKG2D blockade also reduced the cytotoxicity of activated T cells against cultured mouse oligodendrocytes.

### 3.4 RAE-1

Retinoic acid early precursor transcript-1 (RAE-1) is a family (named *α*-*ε*) of one of the multiple ligands for the NK cell activating receptor NKG2D in mice. It serves as an immunosurveillance system, leading to the exposure of pathological cells to an NK cell-dependent immune response.

The expression of RAE-1 (and MULT1) is induced both constitutively and via the DNA damage response (DDR) pathway caused by the presence of cytosolic DNA during pathological cell conditions, with research focusing on various tumor cells, tumor vasculature, and viral infections. DDR relies on a STING-dependent DNA sensor pathway, and its major transducers are the PI3-kinase-related protein kinases ataxia telangiectasia mutated (ATM), ATM and Rad3-related (ATR) and checkpoint kinase-1 homolog (CHK1) (Djelloul et al., 2016; Zhang and Sentman, 2013; Lam et al., 2014).

Several myeloid cells that are activated during EAE express RAE-1, which participates in EAE in a complex and multidimensional manner. Microglial proliferation and activation are hallmark traits of EAE and it is suspected that RAE-1 is a marker of microglial proliferation. Peritoneal macrophages also have the ability to express RAE-1, which is upregulated by Toll-like receptor signaling through the MyD88 adaptor and is thus found on the surface of activated, but not resting, macrophages. RAE-1 can then be detected by NKG2D on NK cells, resulting in downregulation of the receptor. Moreover, myeloid-derived suppressor cells (MDSCs) which are found in the spinal cord during EAE in mice, express RAE-1 (Hasan et al., 2005). MDSCs cooperate with invariant NK T cells to protect the CNS against EAE (Hamerman et al., 2004).

RAE-1 is also linked to cell proliferation. In the CNS, it is physiologically highly expressed in neural precursor cells; it has been demonstrated that the expression persists in the subventricular area, where stem and progenitor cells are still present. The concentration of RAE-1 decreases during differentiation into either neurons or glia; therefore, it is thought to play an additional role during development as a proliferation regulator (Heyward et al., 2012).

EAE resulting from intracranial injection of the neurotropic JHM strain of mouse hepatitis virus was used to show clinical and histological improvements in the intraspinal transplantation of syngeneic neural precursor cells. However, the benefits of allogeneic transplantation are limited owing to MHC mismatch, which results in rapid rejection of the neurograft driven by both the innate immune system and T cells. Weinger et al. (2014) showed that in a virus-induced demyelination model, the NK cell-mediated killing of allogeneic cells is highly dependent on the presence of the RAE-1 receptor, suggesting that the modulation of NKG2D-RAE-1 signaling may be necessary for the long-term survival of CNS allografts.

Some viruses such as murine CMV encode proteins that downregulate the expression of RAE-1 and other NKG2DLs to avoid NK cell-dependent attenuation (O'Callaghan et al., 2001; Kriegeskorte et al., 2005).

### 3.5 H60

Histocompatibility 60 (H60) is a family of MHC class I-like murine cell surface glycoproteins that consists of three members: H60a, H60b, and H60c, all of which function as ligands for NKG2D receptors and share many similarities with RAE-1.

H60 expression on the cell surface is almost undetectable in normal tissues, but can be induced by a variety of different forms of cell stress and tumor cells (Samarakoon et al., 2009). The affinity of NKG2D for both murine ligands was high, yet for H60, it was approximately 25-fold higher than its affinity for binding RAE-1 (O'Callaghan et al., 2001). H60 can also suppress T cell proliferation; however, this response is dependent on the presence of IL-10 (Kriegeskorte et al., 2005).

It was proven by Galazka et al. (2014) that complexes of heat shock proteins (HSPs), which are efficient chaperones with many peptides, play an important role in immunomodulation; one of these complexes (HINT138–57/Hsp70) is able to prevent EAE by inducing immunoregulatory mechanisms dependent on NK cells via their two receptors, CD94 and NKG2D. The HINT138–57/Hsp70 complex also enhances the expression of H60. Downstream signaling of CD94 and NKG2D converges at the adaptor proteins DAP10 (a molecule controlling the activation threshold of autoreactive T cells) and DAP12, which are then upregulated, resulting in NK cells serving as regulatory cells for autoimmune T cells and mediating B cell autoimmunity, which can effectively prevent the development of EAE. Hsp70 complexed with peptides isolated from EAE brains (Hsp70-pc) induced NK cell-dependent resistance to subsequent EAE induction in SJL/J mice, likely due to upregulation of the NKG2D ligand, H60. NKG2D–H60 interactions have been described as potentially capable of modulating dendritic cell function, leading to the elimination of antigen-reactive T cells and the induction of EAE tolerance. This was suggested by the reduced ability of dendritic cells pre-incubated with NK cells from Hsp70-pc mice to stimulate the proliferation of proteolipid protein-reactive cells *in vitro*, which was also correlated with increased proteolipid protein-reactive cell death (Galazka et al., 2007). It was also shown that contact hypersensitivity partially depends on allergen-induced upregulation of NKG2DLs on keratinocytes, which subsequently interact with NKG2D on dendritic epidermal T cells, playing a significant role in the development of allergic contact dermatitis (Nielsen et al., 2015).

### 3.6 Future perspectives and difficulties in targeting NKG2D ligands and its pathways as therapeutic targets for MS

**Figure 2:**
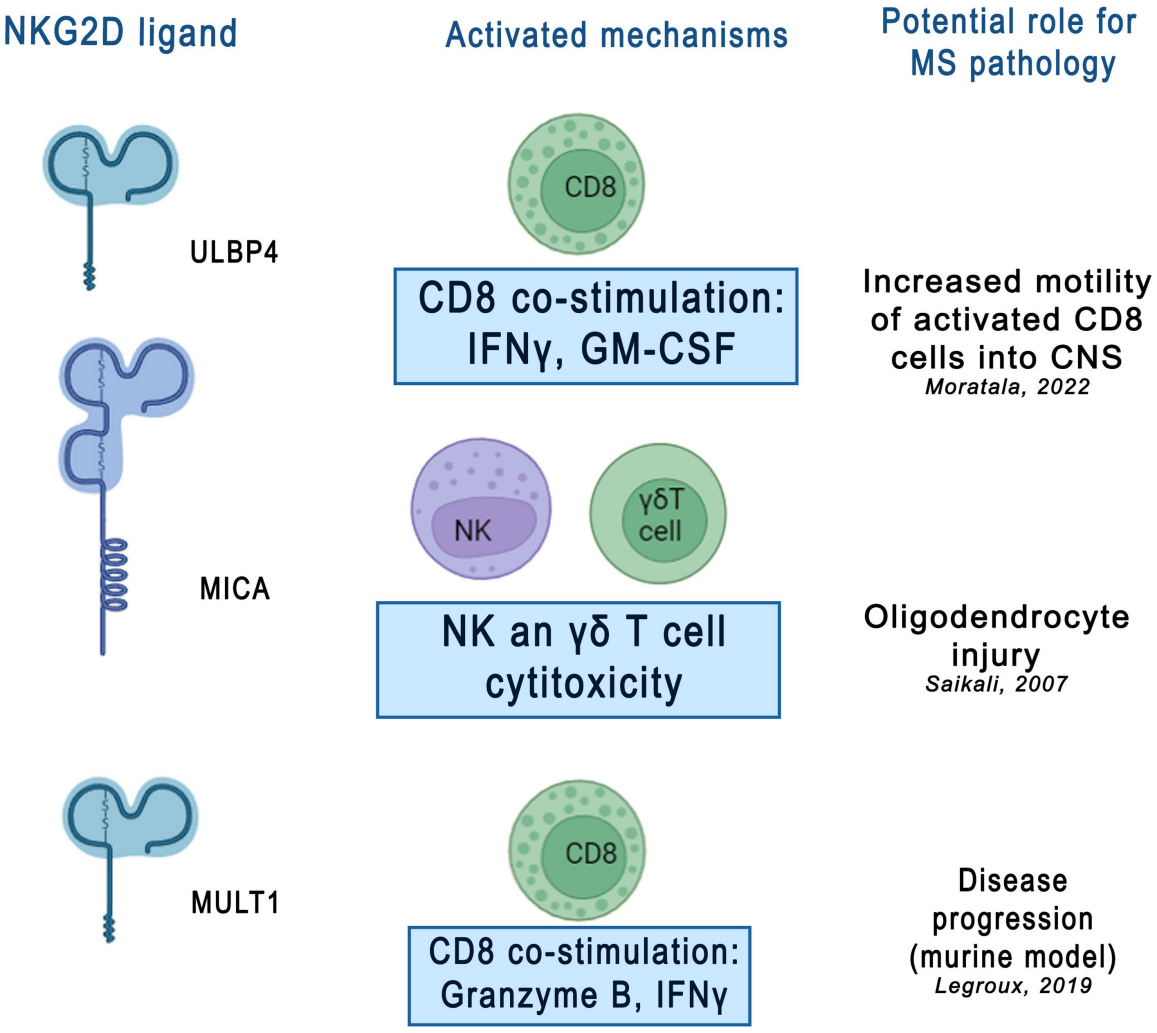
Potential role of NKG2D ligands in multiple sclerosis (MS) pathology. NKG2D ligands, including ULBP4, MICA, and MULT1, were shown activate various immune mechanisms contributing to neuroinflammatory and neurodegenerative processes in MS. This figure was created using Adobe Photoshop. CD8 - cluster of differentiation 8, cytotoxic T lymphocytes, IFNy- interferon gamma, GM-CSF - granulocyte[1]589 macrophage colony-stimulating factor, γδT cell- gamma delta T cells, ULBP4 - UL16-binding protein 4, MICA - MHC 590 class I polypeptide–related sequence A, MULT1- murine UL16 binding protein-like transcript.

Regarding the use of the described proteins as therapeutic options, in the available reports on experimental animal models of EAE, the inhibition of NKG2D + CD4+ T cell migration to the CNS and attenuation of the killing effect of murine oligodendrocytes were possible by blocking the NKG2D pathway (Ruck et al., 2013). It has been also observed that sULBP4 can affect the function of CD8+ T cells, for example by increasing the production of proinflammatory cytokines, GM-CSF and IFN-*γ* and promoting the motor capacity of CD8+ T cells, which was confirmed in EAE models (Legroux et al., 2019; Moratalla et al., 2022). Moreover, after the NKG2D blockade, an increase in the number and motility of CD8+ T cells co-cultured with astrocytes expressing NKG2DL was observed, suggesting that NKG2D is involved in the interaction between CD8+ T cells and astrocytes (Moratalla et al., 2022). In addition, significant reduction of clinical symptoms and regression of pathological changes in EAE induced by myelin oligodendrocyte glycoprotein after NKGD blockade was demonstrated. In addition, the cytotoxicity of activated T cells against cultured mouse oligodendrocytes was reduced by the NKG2D blockade. Similarly, interactions with NKG2D may be an alternative mechanism by which NK cells suppress autoreactive T cells (Galazka et al., 2006). On the other hand, NK cells can also interact directly with brain-resident cell types; upon activation, they can kill resting microglia *in vitro* via NKG2D- and NKp46-dependent pathways (Lünemann et al., 2008). Furthermore, the inappropriate expression of NKG2D and its ligands can lead to the activation of autoreactive effector cells. Additional evidence suggests that NKG2D-expressing NK cells or T lymphocytes contribute to tissue damage in MS by killing NKG2D ligand-bearing oligodendrocytes or astrocytes (Saikali et al., 2007). This suggests that the impact on the NKG2D pathway could prove to be an important therapeutic target in MS. NKG2D interaction with their ligands in MS is shown in Figure 2. However, the limitation of this treatment strategy seems to be primarily because elevated levels of soluble forms of NKG2DLs have been detected in other inflammatory and autoimmune diseases, as described above. Therefore, the selection of a highly selective molecule that can affect NKG2DLs and their receptors closely related to MS is crucial. On the other hand, treatments that target the inflammatory process in a general context could serve as a complement to existing or ongoing therapeutic options targeting other pathophysiological pathways in MS, which could create the possibility of more effective combined treatment. Moreover, there are already trials of therapies targeting the NKG2D pathway – in a randomized, double-blind, parallel, placebo-controlled trial (NCT01203631), the anti-NKG2D antibody NNC0142-0002 showed clinical efficacy in patients with CD, especially in patients treated with new biologics (Giuffrida and Di Sabatino, 2020). At present, the clinical treatment of autoimmune diseases mainly relies on glucocorticoids and immunosuppressants, whereas the expression of NKG2D and NKG2DLs is regulated by many factors. Several targeted antibodies can be developed to block the expression of NKG2D or NKG2D-L and inhibit their interaction.

## 4 Conclusion

The aim of this review was to present and summarize the latest knowledge on NKG2DL family proteins. It has been shown that several cellular stressors present in chronic inflammatory and autoimmune diseases increase the expression of specific NKG2DLs, which consequently leads to the activation of NKG2D-bearing immune effector cells. NKG2DLs alert the immune system when tissue homeostasis is threatened by various conditions such as tumors, infections, inflammation, and cellular stress. Because immune effector cells express the NKG2D receptor under normal physiological conditions, tight regulation of ligand expression is crucial for controlling activation of the NKG2D-NKG2DL pathway. NKG2DLs are subjected to transcriptional, post-transcriptional, translational, and post-translational regulatory mechanisms that modulate their expression. The results of studies presented in this article indicate that pathologically elevated NKG2DL levels may contribute to chronic inflammatory and autoimmune diseases. Some of the aforementioned studies have also confirmed that the levels of soluble forms of NKG2DL proteins are higher in patients with MS. This may indicate their potential role as biomarkers of the inflammatory process in this disease. However, further studies are required to fully understand the exact involvement of these proteins in the pathogenesis of MS.
